# Detection of Anti-*Echinococcus granulosus* Antibodies in Humans: An Update from Pakistan

**DOI:** 10.3390/pathogens11010029

**Published:** 2021-12-28

**Authors:** Huma Khan, Haroon Ahmed, Muhammad Sohail Afzal, Usman Ayub Awan, Muhammad Khurram, Sami Simsek, Jianping Cao

**Affiliations:** 1 Department of Biosciences, COMSATS University Islamabad (CUI), Islamabad 45550, Pakistan; hkhan@hotmail.com; 2 Department of Life Sciences, School of Science, University of Management & Technology (UMT), Lahore 54700, Pakistan; sohail.ncvi@gmail.com (M.S.A.); mohammadkhurram9494@gmail.com (M.K.); 3 Department of Medical Laboratory Technology, The University of Haripur, Haripur 22620, Pakistan; usman.ayub111@gmail.com; 4 Department of Parasitology, Faculty of Veterinary Medicine, University of Firat, Elazig 23119, Turkey; ssimsek@firat.edu.tr; 5 National Institute of Parasitic Diseases, Chinese Center for Disease Control and Prevention (Chinese Center for Tropical Diseases Research), NHC Key Laboratory of Parasite and Vector Biology, WHO Collaborating Centre for Tropical Diseases, National Center for International Research on Tropical Diseases, Shanghai 200025, China; 6 The School of Global Health, Chinese Center for Tropical Diseases Research, Shanghai Jiao Tong University School of Medicine, Shanghai 200025, China

**Keywords:** cystic echinococcosis, hydatid cysts, seropositivity, ELISA, IHA, Pakistan, hydatic cysts

## Abstract

Human cystic echinococcosis (CE) is a zoonotic disease caused by the larval stage of *Echinococcus granulosus* sensu lato that causes economic losses by affecting livestock and also poses a public health threat worldwide. The present study is the first retrospective report on the seroprevalence of anti-*E. granulosus* antibodies in humans in Pakistan. The study used data from 93 blood analysis reports of patients suspected of having CE from different medical centers in Lahore, Pakistan. Out of 93 sera samples, 20 (21.5%) were seropositive, and higher seropositivity (17.2%) was recorded with the indirect hemagglutination test (IHA) than with enzyme-linked immunosorbent assay (ELISA). The findings indicated that age, gender, and year had no significant relationship with the seropositivity of CE. The current study provides directions towards the management of the disease in the near future in Pakistan.

## 1. Introduction

Cystic echinococcosis (CE) is an essential parasitic zoonotic disease in humans and livestock caused by larvae of the cestode parasite *Echinococcus granulosus* sensu lato [1]. The life cycle of the parasite comprises two hosts: the definitive carnivorous host (dogs, wolves, and foxes) in which the adult form of the parasite develops, and an herbivorous or omnivorous host in which the metacestode (larval stage) occurs [2]. Exposure to parasite eggs, excreted into the definitive host feces, plays a significant role in disease transmission [3]. Humans are incidental intermediate hosts and are infected by ingesting parasite eggs via contaminated food and water [4,5]. The metacestode form, which is called the hydatid cyst, develops and causes CE in intermediate hosts such as livestock, and humans behave as accidental intermediate hosts [6]. Intermediate hosts (mostly ungulates and lagomorphs) are the primary reservoirs for human CE infection [7,8].

CE can be asymptomatic for months or years until a large cyst is formed. Cyst formation usually occurs in the liver and lungs but can develop in other organs [9]. The World Health Organization (WHO) categorizes hydatid cysts into three stages: stage CE1 and CE2 (active cyst), stage CE3 (translational cyst), and stage CE4 and stage CE5 (inactive and degenerative cyst) [10]. As CE infection remains asymptomatic for years before the cyst enlarges and causes symptoms, clinical diagnosis is difficult [11]. The mainstay of CE diagnosis is imaging techniques involving ultrasound (US), radiography, computerized tomography (CT), and magnetic resonance imaging (MRI) [7,8,11]. However, these techniques produce data that are hard to interpret and are relatively complex, and small cysts in the preliminary stages are not easily detectable with radiological procedures [12]. Moreover, these imaging technologies are not always available in developing countries with inadequate medical facilities [13,14].

On the other hand, serological tests are more readily available and helpful for providing data on *E. granulosus* presence and detecting asymptomatic cyst carriers. Hence, CE diagnosis is mainly based on imaging techniques, while serological tests are employed as confirmatory tests that detect *E. granulosus* antibodies such as IgG and IgM [7]. With a combination of serological and imaging assessments, individuals can be diagnosed and treated before severe manifestation of the disease [15]. Accordingly, it is recommended that population screening in endemic regions include both serological and US examinations [16].

CE is a cosmopolitan helminthic disease that occurs worldwide over a wide range of geographic areas associated with livestock rearing, such as Australia, eastern and southern Europe, Asia, South America, and the Middle East, and is endemic in many parts of the world, including Pakistan. Multiple socio-cultural and economic factors, as well as poor hygiene practices, are associated with higher transmission and prevalence rates of CE [17,18,19], for example, illiteracy, farming, having pets (especially dogs), and living in rural areas [20]. The prevention and control of CE are essential, as it is associated with loss of livestock and considerable economic losses. In the United States (US), annual losses of USD 3 billion were estimated to occur due to loss of wages, treatment costs, and production losses associated with livestock [21]. Further, the direct cost associated with the surgical management of CE patients amounted to USD 4,068,666, with USD 3,951,853 attributable to direct diagnosis- and treatment-related costs, and USD 117,137 attributable to wage losses during the treatment period [22]. Each year, it is predicted that 188,000 people worldwide contract E. granulosus, resulting in the loss of 184,000 disability-adjusted life years (DALYs) [23]. Pakistan is considered an endemic CE region [24], but the disease burden has been poorly studied in Pakistan [17]. Thus, little is known about the disease’s epidemiology and its importance in public health [25].

To the best of our knowledge, no comprehensive sero-epidemiological study on human CE has been conducted in Pakistan, even though there are records of CE in several hospitals in the country. Therefore, this study aimed to evaluate the seropositivity of human CE in Pakistan.

## 2. Results

**Figure 1:**
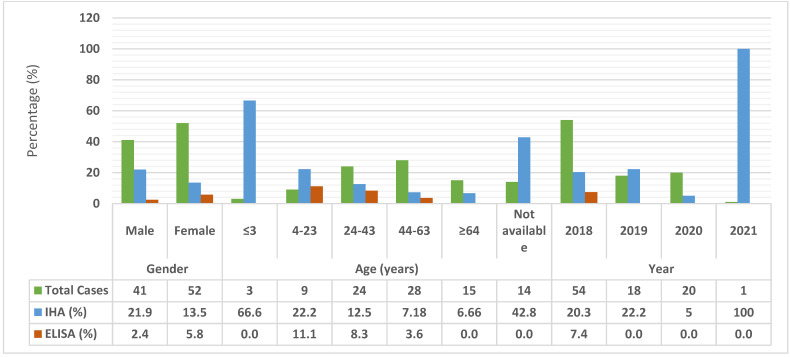
Seroprevalence of human cystic echinococcosis according to gender, age, and year of testing.

Out of the 93 cases included in this study, the majority (54, 58.0%) of the cases were recorded in 2018 and 20 (21.5%) were recorded in 2020. Concerning the distribution of cases according to age group, the highest number of cases (28, 30.1%) was observed in the age group 44–63 years, followed by the age group 24–43 years (24, 25.8%). Of the 93 patients, 52 (55.9%) were females and 41 (44.1%) were males. Further, 20 (21.5%) patients were found to be seropositive with a titer level of >12 and 71 (77.4%) were negative (<9), according to both the commercially available enzyme-linked immunosorbent assay (ELISA) and indirect hemagglutination (IHA) test results (Table 1). The highest prevalence was recorded using the IHA test, with seropositivity of 21.9% in males and 13.5% in females; for ELISA, the seropositivity recorded in females was high at 5.76%, compared to 2.43% in males (Figure 1).

The association of age, year, and gender with anti-*E. granulosus* antibodies was detected using ELISA and IHA testing. According to the results of ELISA, seroprevalence was the highest in the age group 24–43 years (n = 2) and, according to the IHA results, too, the highest seroprevalence was recorded in the age group 24–43 years (n = 3), followed by the age group 44–63 years (n = 2). However, the difference in seroprevalence between the age groups was not statistically significant (χ^2^ = 1.58, *p* = 0.811) (Table 2). Gender-wise distribution showed that there was a higher number of seropositive cases among females (n = 3) than among males (n = 1) according to the ELISA results, while a higher prevalence was recorded in males (n = 9) than in females (n = 7) according to the IHA results. The overall prevalence of CE was similar in both sexes (χ^2^ = 1.25, *p* = 0.264). Most cases were recorded in 2018, according to the results of both ELISA (n = 4) and IHA (n = 7) (Table 2). However, there was no significant association between year and CE prevalence (χ^2^ = 1.66, *p* = 0.435).

## 3. Discussion

Cystic echinococcosis is an emerging disease with a worldwide geographic distribution and is endemic in many parts of world, including Pakistan. In Pakistan, limited studies are available on human CE by using serological tests (ELISA/IHA) to rule out the epidemiology of disease and its public health importance [1,26,27,28,29]. To the best of our knowledge, the current report is the first retrospective study on the seropositivity of human CE in Pakistan. The seroprevalence across several hospitals was determined using ELISA and IHA. Previous reports have shown that the sensitivity of the ELISA and IHA tests performed for the serodiagnosis of 57 surgically confirmed human cases was 91.2% and 68.4%, respectively [30,31]. Alternatively, IHA is an easy-to-use sero-diagnostic test with high sensitivity and specificity [32]. Some studies have shown that ELISA and IHA are complementary and have similar sensitivity [3,33]. Therefore, both ELISA and IHA data were used in this study.

In the current study, the overall seropositivity of human CE was calculated as 21.5%. In comparison, the seroprevalence recorded in the Mediterranean basin region of Greece by Sotiraki et al. (2003) [34] was higher at 29.00%, although Andrabi et al. (2020) found a seroprevalence rate that was 4.4% lower than the present seropositivity rate in south Kashmir, India [4,8]. Further, Aklani et al. (2014) [35] also recorded the seropositivity rate of 6.9% in four different towns of Denizli, Turkey. Numerous serological assays have been used with various antigens, each with its own set of limitations, both test- and antigen-dependent [11,36]. Numerous medical facilities rely on ELISA due to its widespread availability, especially in Pakistan. In our recent review, we found that 28 (58.3%) of the publications (having the majority of the case reports) included the use of serology to diagnose CE, with indirect hemagglutination (IHA) being the most often employed approach. No study reported the use of immunoblotting. Recently, it was proposed that this assay is the most reliable single confirmatory test for abdominal CE [37]. For the diagnosis of CE, sixteen publications (33.3%) used more than one serological test. Increased specificity can be achieved by using more than one test panel. No immunochromatographic tests were used in any of the 30 studies that observed patients after surgery was completed. In hospital-based studies, these fast, point-of-care tests have been proven to perform as well as ELISA [38,39,40]. However, they are still being validated in resource-constrained environments.

CE affects people of almost all ages, from below 3 to above 80 years [8], but CE infection generally increases with age. Our study recorded the highest seropositivity rate in participants aged 31–50 years (5.3%), followed by the age group 51–70 years. Individuals of this age group may be more likely to be exposed to *E. granulosus-*contaminated environments. Another reason might be that CE remains asymptomatic for years and is considered to be a slow-growing chronic disease. The findings of this study are in accordance with previous studies, which have reported prevalence rates of 47.8% and 46.7% in the age group 20–40 years [41,42]. In contrast, other studies have reported that the prevalence peaked in the age group 30–60 years [15,35], and Adrabi et al. (2020) [8] reported more seropositive cases in the 1- to 17-year-old age group. In this study, the 1–17 age group was more prone to be seropositive across various occupations in all four districts of south Kashmir that may be more likely to be exposed to echinococcus-infected dogs and an infected environment [8].

In this study, no sex-dependent, statistically significant difference was detected, as both males and females were found to be equally positive for CE. This is possible because most people in Pakistan reside in rural regions linked with the agricultural sector, where both males and females are equally active in farming and livestock raising and come into contact with dogs and infected food during production.

The major limitation in our study is the small sample size, as only individuals who had specimens taken for histopathological examination and records available were included. Therefore, the current report’s data analysis probably underestimates the incidence of the CE seropositivity rate in Pakistan.

## 4. Material and Methods

### 4.1. Study Area

**Figure 2:**
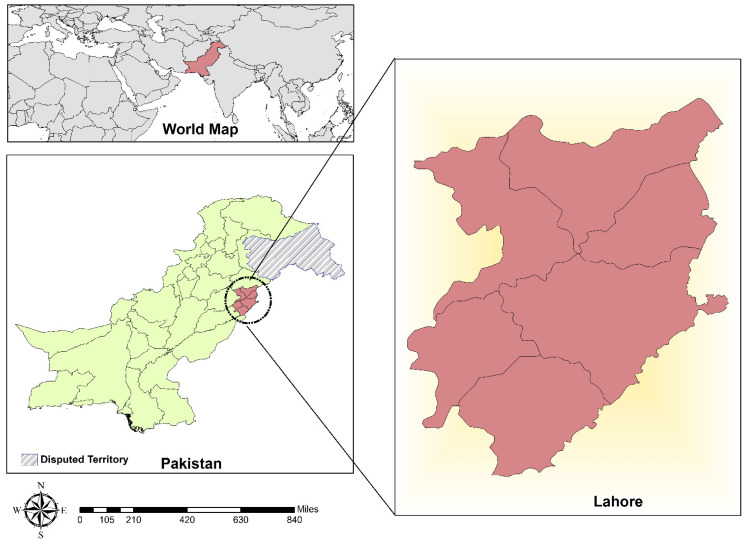
Study area map of Lahore city.

The present study was conducted in Lahore, Punjab province, Pakistan. Punjab is one of the largest provinces by population, with fertile agricultural land and deserts in the southern part neighboring Rajasthan and the Sulaiman Range. Lahore is the capital city of Punjab and is a pivotal part of the country’s cultural diversity (Figure 2). According to the current census, the second-most populous city of Pakistan is Lahore, with 40% of its inhabitants being ≤ 15 years of age and a literacy rate of only 64%. The study area has diverse environmental conditions, with extreme summers and moderate winters [43].

### 4.2. Methods

The current study included 93 serological test reports of anti-*Echinococcus granulosus* antibodies in patients suspected of being infected from different serodiagnostic centers between 2018 and 2021 in Lahore, Pakistan. ELISA and IHA tests were used to detect anti-*E. granulosus* antibodies in the serum samples. The recorded data included the patient’s age, gender, and serum test performed for detecting anti-*E. granulosus* antibodies.

Echinococcus antibodies were detected using the DRG Echinococcus IgG ELISA Kit (ECHG0130BA) as per manufacturer guidelines. Microtiter wells were coated with antigen as a solid phase. These wells were used for pipetting diluted patient samples and ready-to-use control samples. The color intensity was determined following the addition of primary and secondary antibodies, and it was shown to be directly related to the amount of echinococcus IgG antibodies in the sample [44]. Similarly, the indirect hemagglutination titer was estimated following the manufacturer’s instructions (ELIHA kit) [45].

### 4.3. Statistical Analysis

Statistical analysis of the data was performed using SPSS 18.0 for Windows. The chi-square test with a 5% significance level was used to assess the association between seropositivity for anti-*E. granulosus* antibodies and each risk factor. The results were considered statistically significant when the *p*-value was below 0.05.

## 5. Conclusions

CE is widespread in Pakistan and is a significant health concern in the country. According to the current study’s research, the seropositivity of human CE in Pakistan exhibited no significant association with age, year, or gender. The current study is the first retrospective report on the seropositivity of human CE in a population-based setting. Furthermore, no prior investigation on this topic has been reported in Pakistan; hence, the current data serve as a baseline for future monitoring of human CE infection and may aid in designing control measures in Pakistan.

## Figures and Tables

**Table 1:** Seroprevalence (IHA and ELISA) of hydatid disease according to year, age, and gender.

Variables		Total	IHA			ELISA			
			Positive	Negative	Prevalence (%)	Positive	Negative	Elevated	Prevalence (%)
Gender	M	41	9	22	21.9	1	9	0	2.43
	F	52	7	25	13.5	3	16	1	5.76
Age	≤3	3	2	1	66.6	0	0	0	0
	4–23	9	2	4	22.2	1	2	0	11.11
	24–43	24	3	7	12.5	2	11	1	8.33
	44–63	28	2	19	7.18	1	6	0	3.57
	>64	15	1	8	6.66	0	6	0	0
	Not available	14	6	8	42.8	0	0	0	0
Year	2018	54	11	14	20.3	4	24	1	7.40
	2019	18	4	13	22.2	0	1	0	0
	2020	20	1	19	5.00	0	0	0	0
	2021	1	1	0	100	0	0	0	0

**Table 2:** Chi-square analysis of multiple factors associated with seropositivity for echinococcosis.

Variable	Category	ELISA, n (%)		IHA, n (%)		Chi-Square	*p*-Value
Age (y)	≤3	0 (0%)	0	2 (100%)	100	1.58	0.811
	4–23	1 (33.3%)	33.3	2 (66.7%)	66.7		
	24–43	2 (40.0%)	40.0	3 (60.0%)	60.0		
	44–63	1 (33.3%)	33.3	2 (66.7%)	66.7		
	>64	0 (0%)	0	1 (100%)	100		
	Not available	0 (0%)	0	6 (100%)	100		
Gender	F	3 (30.0%)	30.0	7	70.0	1.25	0.264
	M	1 (10.0%)	10.0	9	90.0		
Year	2018	4 (26.7%)	26.7	11 (73.3%)	73.3	1.66	0.435
	2019	0 (0%)	0	4 (100%)	100		
	2020	0 (0%)	0	1 (100%)	100		

## Data Availability

Not applicable.
